# Sirtuin 3 regulation: a target to alleviate β-hydroxybutyric acid-induced mitochondrial dysfunction in bovine granulosa cells

**DOI:** 10.1186/s40104-022-00825-w

**Published:** 2023-02-14

**Authors:** Shanjiang Zhao, Jianfei Gong, Yi Wang, Nuo Heng, Huan Wang, Zhihui Hu, Haoyu Wang, Haobo Zhang, Huabin Zhu

**Affiliations:** State Key Laboratory of Animal Nutrition, Key Laboratory of Animal Genetics, Breeding and Reproduction of Ministry of Agriculture and Rural Affairs, Institute of Animal Science, Chinese Academy of Agricultural Sciences, Beijing, China

**Keywords:** BHBA, Dairy cows, Granulosa cells, Ketosis, Mitochondrial function, Sirt3

## Abstract

**Background:**

During the transition period, the insufficient dry matter intake and a sharply increased in energy consumption to produce large quantities of milk, high yielding cows would enter a negative energy balance (NEB) that causes an increase in ketone bodies (KBs) and decrease in reproduction efficiency. The excess concentrations of circulating KBs, represented by β-hydroxybutyric acid (BHBA), could lead to oxidative damage, which potentially cause injury to follicular granulosa cells (fGCs) and delayed follicular development. Sirtuin 3 (Sirt3) regulates mitochondria reactive oxygen species (mitoROS) homeostasis in a beneficial manner; however, the molecular mechanisms underlying its involvement in the BHBA-induced injury of fGCs is poorly understood. The aim of this study was to explore the protection effects and underlying mechanisms of Sirt3 against BHBA overload-induced damage of fGCs.

**Results:**

Our findings demonstrated that 2.4 mmol/L of BHBA stress increased the levels of mitoROS in bovine fGCs. Further investigations identified the subsequent mitochondrial dysfunction, including an increased abnormal rate of mitochondrial architecture, mitochondrial permeability transition pore (MPTP) opening, reductions in mitochondrial membrane potential (MMP) and Ca^2+^ release; these dysfunctions then triggered the caspase cascade reaction of apoptosis in fGCs. Notably, the overexpression of Sirt3 prior to treatment enhanced mitochondrial autophagy by increasing the expression levels of Beclin-1, thus preventing BHBA-induced mitochondrial oxidative stress and mitochondrial dysfunction in fGCs. Furthermore, our data suggested that the AMPK-mTOR-Beclin-1 pathway may be involved in the protective mechanism of Sirt3 against cellular injury triggered by BHBA stimulation.

**Conclusions:**

These findings indicate that Sirt3 protects fGCs from BHBA-triggered injury by enhancing autophagy, attenuating oxidative stress and mitochondrial damage. This study provides new strategies to mitigate the fGCs injury caused by excessive BHBA stress in dairy cows with ketosis.

**Supplementary Information:**

The online version contains supplementary material available at 10.1186/s40104-022-00825-w.

## Introduction

In a variety of conditions, such as fasting and uncontrolled diabetes, there is an increase in the concentration of circulating ketone bodies (KBs) in the blood. Appropriate concentrations of KBs are considered to be beneficial in most cases because they represent an effective source of energy to fuel the body’s energy needs [1, 2]. Yet, when KBs concentrations are abnormally elevated, the role is reversed from “friend” to “enemy”; this causes various pathological complications by activating detrimental pathways [3]. Moreover, most high yielding cows are prone to undergo negative energy balance (NEB) after calving; this causes excessive fat mobilization and fatty acid overload in the liver, ultimately producing an excess of KBs being released into the circulation [4, 5]. The KBs include β-hydroxybutyric acid (BHBA), acetoacetic acid and acetone; BHBA is the most stable and accounts for approximately 70% of all KBs. A BHBA concentration exceeding 1.2 mmol/L is diagnosed as subclinical ketosis and can exert subsequent negative effects on reproductive performance (e.g., prolonging the first estrus period and the first insemination period) [6, 7]. A previous study showed that metabolites between the serum and follicular fluid (FF) are strongly correlated and that increased concentrations of BHBA in the serum are also seen in FF, which may affect both granulosa cells and oocyte quality [8, 9]. However, the mechanisms by which BHBA affects follicular granulosa cells (fGCs) and follicle development remain unclear.

β-Hydroxybutyric is an intermediate product of fatty acid oxidation (FAO) and is crucial for the regulation of energy homeostasis [10]. However, excessive concentrations of BHBA have been shown to be associated with oxidative stress and the inhibition of follicular development in ketotic cows [11, 12]. Similarly, high concentrations of BHBA entering the follicular microenvironment may cause oxidative stress and inhibit follicular development. Furthermore, during the development of the follicle from the primordial follicle to the corpus luteum, fGCs secrete steroid hormones, growth factors and cytokines that are crucial for oocyte quality and follicle development [13]. The abnormal apoptosis of fGCs may trigger abnormal follicular atresia, thus leading to a reduction in follicle numbers and ultimately reduced fertility [14]. Recent studies demonstrated that oxidative stress induced by different factors is the primary factor driving apoptosis in fGCs [15–18]. Increased reactive oxygen species (ROS) synthesis due to oxidative stress normally occurs in the mitochondria and could trigger a reduction in mitochondrial membrane potential (MMP) and release pro-apoptotic mediators to promote programmed cell death [14, 19–21]. Therefore, we hypothesize that BHBA overload may disrupt mitochondrial redox homeostasis and cause dysfunction of mitochondria, thus causing cellular injury.

Sirtuin 3 (Sirt3) is a nicotinamide adenine dinucleotide (NAD^+^)-dependent deacetylase in mitochondria and plays an integral role in mitochondrial oxidative metabolism, energy metabolism, signaling regulation and apoptosis [22, 23]. The knockdown of Sirt3 in vitro [24, 25] and in vivo [26, 27] demonstrated that Sirt3 decrease leads to severe mitochondrial dysfunction and oxidative stress. Conversely, increased levels of Sirt3 can ameliorate mitochondrial damage and oxidative stress, which in turn protects against apoptosis [28–32]. In addition, studies have indicated that Sirt3 deacetylation could activate the downstream AMPK pathway involved in energy metabolism [33, 34]. AMPK could interact with mTOR to regulate biological processes involving energy consumption, such as cell growth and cell autophagy [35–37]. These lines of evidence indicate that Sirt3 is a promising target for alleviating BHBA-induced cell mitochondrial oxidation injury.

Herein, we proposed a hypothesis that under BHBA overload-stress, Sirt3 may preserve redox homeostasis as a feedback mechanism. To test our hypothesis, the present study preliminarily confirmed that the fGCs exposed to high concentrations of BHBA, would suffer from oxidative injury and mitochondrial dysfunction, and ultimately to show a greater apoptosis rate. Further experiments revealed the mechanism of Sirt3 overexpression in alleviating BHBA overload-induced mitochondrial oxidative damage. Additionally, Sirt3-regulated protective mechanisms may be related to the enhanced autophagic signaling pathway.

## Methods

### Ethics statement

All of the animal procedures used in this study were approved by the Animal Care and Use Committee of the Institute of Animal Sciences of Chinese Academy of Agricultural Sciences. The approval number is IAS2021-19.

### Cell collection, culture and treatment

Ovaries from cows were collected from the slaughterhouse and washed with a 37 °C pre-warmed 0.9% NaCl solution (containing 100 U/mL penicillin and 0.1 mg/mL streptomycin). Healthy follicles of 2–6 mm in diameter, yellowish, clear and without blood contamination were extracted with a 10-mL syringe (KDL, Shanghai, China). The collected follicular fluid (FF) was then filtered into a new 15-mL centrifuge tube (430790, Corning, New York, USA) and washed three times with Dulbecco’s Phosphate-Buffered Saline (DPBS, 14190-144, Gibco, California, USA). The supernatant was discarded after centrifugation at 1500 r/min for 5 min. Then the fGCs were resuspended in pre-warmed DMEM/F12 (11330032, Gibco, California, USA) complete medium containing 10% fetal bovine serum (FBS, 10091148, Gibco, California, USA) and 1% penicillin-streptomycin. Finally, fGCs were seeded into 6-well plates at a concentration of 1.2 × 10^6^ per well and incubated at 37 °C with 5% CO_2_ for 24 h; the media was replaced with fresh media after 24 h.

All fGCs were first pre-cultured in 6-well plates for 48 h before treatment. To create the BHBA stress model, we starved fGCs in FBS-free medium for 12 h and then changed to DMEM/F12 medium with different concentrations of BHBA (H6501-5G, Sigma, Missouri, USA) and 2% FBS for a further 12 h. For RNA interference, samples were collected at 24, 48 and 72 h after siRNA transfection for subsequent assays. For RNA overexpression plasmid transfection, a Sirt3 overexpression plasmid (PEX3-Sirt3) and empty plasmid (PEX3) were transfected into fGCs for 24 h. The fGCs were treated with serum-free starvation for 12 h followed by the continued exposure of fGCs to BHBA for 12 h.

### Immunofluorescence assays

The extracted fGCs were incubated in confocal dishes for 48 h. Cells were fixed with 4% paraformaldehyde for 1 h at room temperature and then permeabilized with 0.5% Triton X-100 for 40 min. Afterwards, fGCs were blocked with DPBS containing 1% BSA at 4 °C overnight. fGCs were then incubated with anti-FSHR-specific antibodies (Table S1) overnight at 4 °C (incubated with DPBS as a negative control). fGCs were then incubated with fluorescein-conjugated Goat Anti-Rabbit IgG at 37 °C for 1 h. The nuclei of fGCs were incubated with DAPI (C1006, Beyotime, Shanghai, China) for 5 min. Representative images were captured by confocal microscopy (Leica, Germany).

### Cell proliferation assays

Cell viability analysis was performed using the Cell Counting Kit-8 (C0037, Beyotime, Shanghai, China). The fGCs were seeded in 96-well plates and treated with BHBA. Then, these treated cells were incubated with 10 µL of CCK-8 solution for 1.5 h at 37 °C. Equal volumes of cell culture medium, BHBA and CCK-8 solution but no cells were used as a blank control. The optical density (OD) of each well at a wavelength of 450 nm was then measured using a microplate reader (Tecan Infinite 200 Pro).

### Protein isolation and western blotting

Total proteins were extracted by RIPA lysis buffer (P0013B, Beyotime, Shanghai, China) at 4 °C. Protein concentration was then measured using an Enhanced BCA Protein Assay Kit (P0010S, Beyotime, Shanghai, China). Protein samples were mixed proportionally with 5 × loading buffer (P06M18, Gene-Protein Link, Beijing, China) and boiled at 100 °C for 5 min. Subsequently, 40 µg of protein was separated by SDS-polyacrylamide gel electrophoresis (SDS-PAGE) and transferred onto a nitrocellulose membrane (HATF00010, Merck-Millipore, Darmstadt, Germany). Membranes were blocked by 5% skimmed milk for 2 h and then incubated with primary antibody (Table S1) at 4 °C overnight. Then, the membranes were incubated with a horseradish peroxidase-conjugated secondary antibody (Table S1) for 2 h at room temperature. Finally, the films were exposed by Chemistar High-signal ECL western blotting substrate (180–501, TANON, Shanghai, China) and the quantitative analysis was carried out with ImageJ software.

### RNA isolation and quantitative real-time polymerase chain reaction (qRT-PCR)

Total RNA was extracted from fGCs using the Cell Total RNA Extraction Kit (DP430, TIANGEN, Beijing, China). Total RNA was reverse transcribed using the PrimeScrip RT reagent Kit with gDNA Eraser (RR047A, TAKARA, Kyoto, Japan). The qRT-PCR assays were then performed using PowerUp™ SYBR™ Green Master Mix (A25742, ABI, Foster City, CA, USA) and a QuantStudio™ 7 Flex System (ABI). The gene expression levels of each gene were calculated by the 2^−ΔΔCT^ method. All primers were designed by using National Center for Biotechnology Information. The primer information was listed in Table S2 and the house-keeping gene *β-Actin* was used as a reference. Each sample was analyzed in triplicate.

### Apoptosis assays

Apoptosis was assessed using the Annexin V-FITC Apoptosis Detection Kit (C1062M, Beyotime, Shanghai, China). Treated cells were collected and resuspended by 195 µL of Annexin V-FITC conjugate; then, 5 µL of Annexin V-FITC and 10 µL of propidium iodide (PI) staining solution were added sequentially and incubated for 15 min at room temperature. Also, unstained cells as a negative control, the FITC and PI single-stained cells as a compensated control. Subsequently, the prepared cells were detected using a flow cytometer (BD FACSVerse, San Jose, CA, USA). Analysis was performed using FlowJo software version V10.

### Reactive oxygen species (ROS) level assays

Cytosolic ROS (cROS) levels were detected using a ROS Assay Kit (S0033S, Beyotime, Shanghai, China). The collected treated cells were suspended in 10 µmol/L DCFH-DA and incubated for 20 min at 37 °C. The stained cells were then detected using a flow cytometer (BD FACSVerse, USA). FlowJo software version V10 was used for analysis.

We used the MitoSOX™ Red mitochondrial superoxide indicator (M36008, Invitrogen, California, USA) to detect mitoROS levels. The treated fGCs were stained with 5 µmol/L MitoSOX™ and incubated for 10 min at 37 °C under light-protected conditions. Then, fGCs were washed three times with DPBS and observed with a confocal microscope (Leica, Germany). The fluorescence intensity was then analyzed by ImageJ software.

### MDA, Mn-SOD and ATP production assays

Malondialdehyde (MDA) levels were assessed using a Lipid Peroxidation MDA Assay Kit (S0131S, Beyotime, Shanghai, China). In brief, cells were lysed with RIPA and the protein concentration was measured using a BCA kit. Then, 100 µL of cell lysates were incubated with 200 µL of MDA test solution at 100 °C for 15 min. Lysates were then centrifuged at 1000 × *g* for 10 min and 200 µL of supernatant was added to a 96-well plate; absorbance was then read at 532 nm using a microplate reader.

A Cu/Zn-SOD and Mn-SOD Assay Kit with WST-8 (S0103, Beyotime, Shanghai, China) was used to determine intracellular Mn-SOD activity. In brief, cell homogenates were obtained using an ultrasound cell breaker and protein concentration was measured using a BCA kit. The samples were treated with a Cu/Zn-SOD inhibitor and then 20 µL of the treated samples, 160 µL of WST-8 test solution and 20 µL of reaction starter were mixed in 96-well plates to incubate at 37 °C for 30 min. Finally, the absorbance reading at 450 nm was determined with a microplate reader.

An Enhanced ATP Assay Kit (S0027, Beyotime, Shanghai, China) was used to assess intracellular ATP levels. Cells were first lysed with RIPA according to the manufacturer’s instructions; then, protein concentrations were determined using a BCA kit. Subsequently, 100 µL of ATP assay solution was added to 96-well white solid plates (3922, Corning, New York, USA) and left at room temperature for 5 min. Then, we added 20 µL of treated sample and mixed. The luminescence value was measured using a microplate reader.

### Transmission electron microscopy (TEM)

The mitochondrial morphology of fGCs was observed by TEM. In brief, treated cells were pre-fixed for 10 min using 2.5% glutaraldehyde and 2% osmium tetroxide at 4 °C and then post-fixed for 1 h using 3% potassium hexacyanoferrate and 2% osmium tetroxide at 4 °C avoiding light. Cells were then dehydrated using acetone and then flat embedded in fresh resin. After dehydration using acetone, the sample was flat embedded in resin. Ultrathin sections of approximately 60 nm were sectioned using a diamond knife and counterstained with uranyl acetate and lead citrate. A HT7700 transmission electron microscope was used to observe and photograph specimens. The number of abnormal mitochondria were then counted using ImageJ software.

### MPTP assay

We used an MPTP Assay Kit (C2009S, Beyotime, Shanghai, China) to detect the degree of MPTP opening. Calcein AM staining solution (Calcein AM), fluorescence quenching solution (Calcein AM + CoCl_2_) and ionomycin control solution (Calcein AM + CoCl_2_ + ionomycin) were prepared according to the manufacturer’s instructions. Then, we incubated the fGCs for 30 min at 37 °C using the pre-prepared solution. Then, we changed to fresh 37 °C pre-warmed culture medium and incubated at 37 °C for 30 min to ensure that the intracellular esterase sufficiently hydrolyzed the calcein AM to produce calcein with green fluorescence. The fGCs were then washed three times with DPBS and the stained cells were observed using an inverted fluorescence microscope (Nikon, Tokyo, Japan). The fluorescence intensity was then analyzed by ImageJ software.

### Intracellular Ca^2+^ assays

We used 5 µmol/L Fluo-4 AM (S1060, Beyotime, Shanghai, China) for intracellular Ca^2+^ staining; cells were incubated for 20 min at 37 °C under light-protected conditions. Subsequently, the cells were washed three times with DPBS and incubated again for 20 min at 37 °C. The stained cells were then observed using a confocal microscope (Leica, Germany). The fluorescence intensity was then analyzed by ImageJ software.

### Mitotracker staining assays

We used 200 nmol/L Mito-Tracker Red CMXRos (C1035, Beyotime, Shanghai, China) for mitochondrial staining; cells were incubated for 30 min at 37 °C under light-protected conditions. DAPI (C1006, Beyotime, Shanghai, China) was then used to stain nuclei in the cells. After washing with DPBS, the stained cells were observed with a confocal microscope (Leica, Germany). The fluorescence intensity was then analyzed by ImageJ software.

### Cell transfection

siRNAs directed against Sirt3 (shown in Table S2) and the Sirt3-overexpression plasmid (PEX3-Sirt3) were obtained from GenePharma company (Shanghai, China). PEX3-Sirt3 was designed according to the GenBank reference sequence (XM_005200933.4), the specific sequences were shown in Fig. S1. We used Lipofectamine 3000 (Invitrogen, California, USA) to transfect Sirt3-siRNA or PEX3-Sirt3 into fGCs according to the manufacturer’s instructions.

### Statistical analysis

All experiments were performed with at least three independent biological replicates and all data are presented as mean ± standard deviation (SD). SAS software version 9.2 was used for statistical analysis. Comparisons between two groups were made using two-tailed unpaired Student *t*-tests. One-way analysis of variance (ANOVA) followed by Duncan’s test for multiple comparison was also applied. The model for ANOVA was followed as ${y}_{ij}={group}_{i}+ {e}_{ij}$, where ${y}_{ij}$ is the measures of the observation, ${group}_{i}$ is a fixed effect of treatment, and ${e}_{ij}$ is the residual effects. A *P* value < 0.05 was considered statistically significant.

## Results

### β-Hydroxybutyric acid -induced apoptosis in bovine fGCs

**Fig. 1:**
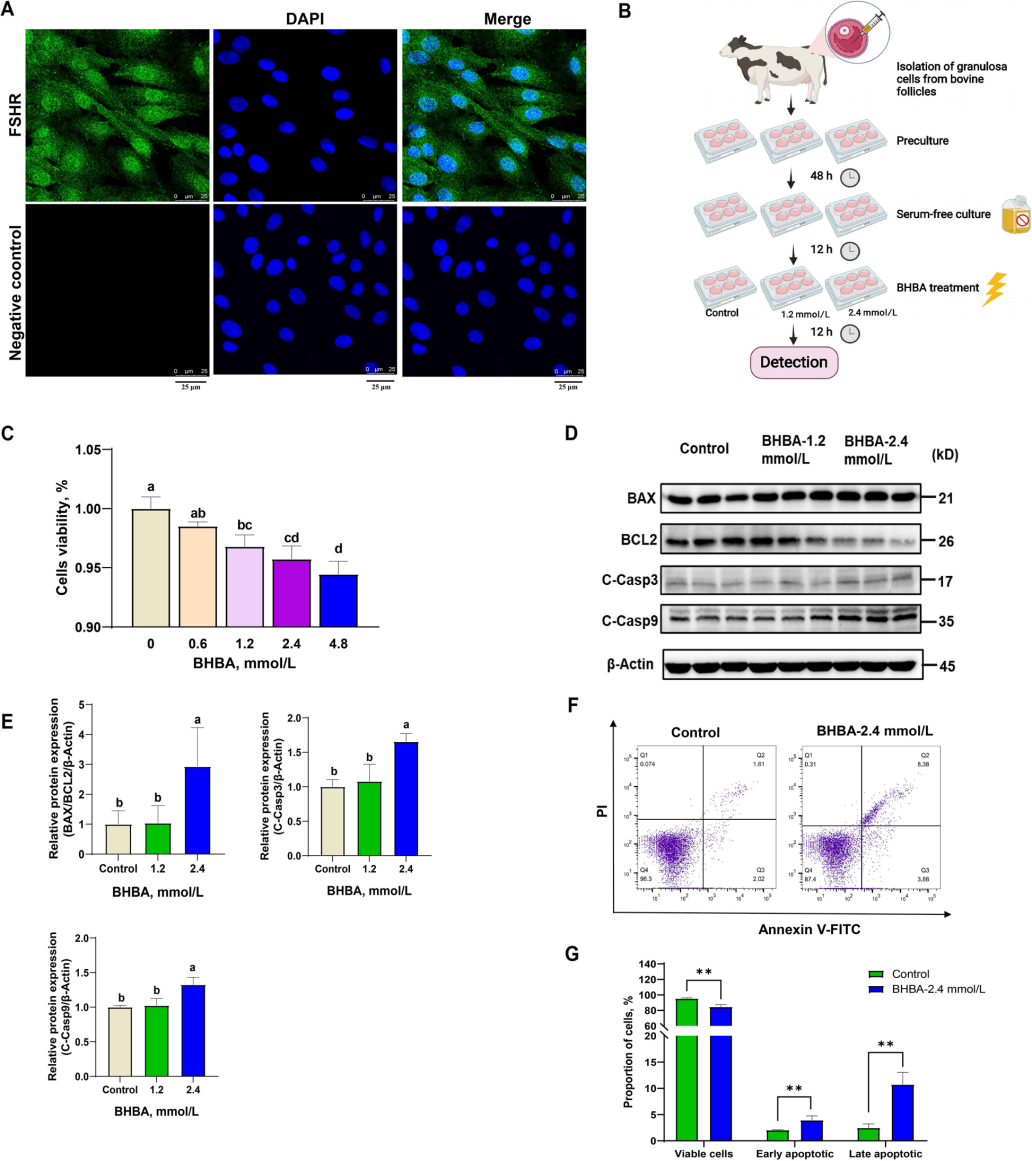
β-Hydroxybutyric acid-induced apoptosis in fGCs. **A** Representative images of FSHR staining in fGCs (scale bars = 25 μm). **B** Schematic protocol for BHBA treatment. **C** Cell viability of fGCs under different concentrations of BHBA stimulation (*n* = 6). **D**–**E** Western blot analysis of BAX, BCL2, cleaved-caspase3, and cleaved-caspase9 in the control, BHBA-1.2 mmol/L and BHBA-2.4 mmol/L groups (*n* = 3). **F**–**G** Apoptosis of fGCs in the control and BHBA-2.4 mmol/L treated groups were analyzed by flow cytometry after stimulation of fGCs with BHBA (*n* = 3). Bar graph showing the proportion of viable cells, early apoptotic cells and late apoptotic cells. For Fig. 1 C and E, we used one-way ANOVA for significant difference analysis (the different lowercases represent *P* < 0.05); for Fig. 1G, we used Student *t*-tests for significant difference analysis (^*^P < 0.05; ^**^P < 0.01)

We isolated fGCs from bovine ovaries and cultured these in vitro. Then, we used immunofluorescence to detect the follicle stimulating hormone receptor (FSHR) and characterize fGCs [38]. Our results showed that the bovine fGCs were of high purity and could be used for subsequent testing (Fig. 1 A).

The effects of BHBA on cells are widely discussed, but, does the increased BHBA affect the development of bovine fGCs? To explore this question, we mimicked ketosis by exposing fGCs to BHBA in vitro (Fig. 1B). We found that the proliferation rate of fGCs exposed to 1.2 mmol/L, 2.4 mmol/L and 4.8 mmol/L BHBA was significantly reduced in a concentration dependent manner when compared to the control group (Fig. 1 C). Furthermore, pro-apoptosis related proteins (BAX/BCL2, cleaved-caspase3 and cleaved-caspase9) were significantly increased in the BHBA 2.4 mmol/L treatment group; however, there was no significant difference at 1.2 mmol/L (Fig. 1D and E). Therefore, 2.4 mmol/L BHBA treatment was used for the subsequent experiments. Consistently, flow cytometry data showed that the proportion of viable fGCs decreased significantly after 2.4 mmol/L BHBA stimulation, while the proportion of cells in early apoptosis and late apoptosis increased significantly (Fig. 1F and G). These results suggested that high concentrations of BHBA (2.4 mmol/L) may trigger apoptosis in bovine fGCs.

### β-Hydroxybutyric acid enhanced peroxidation in bovine fGCs

**Fig. 2:**
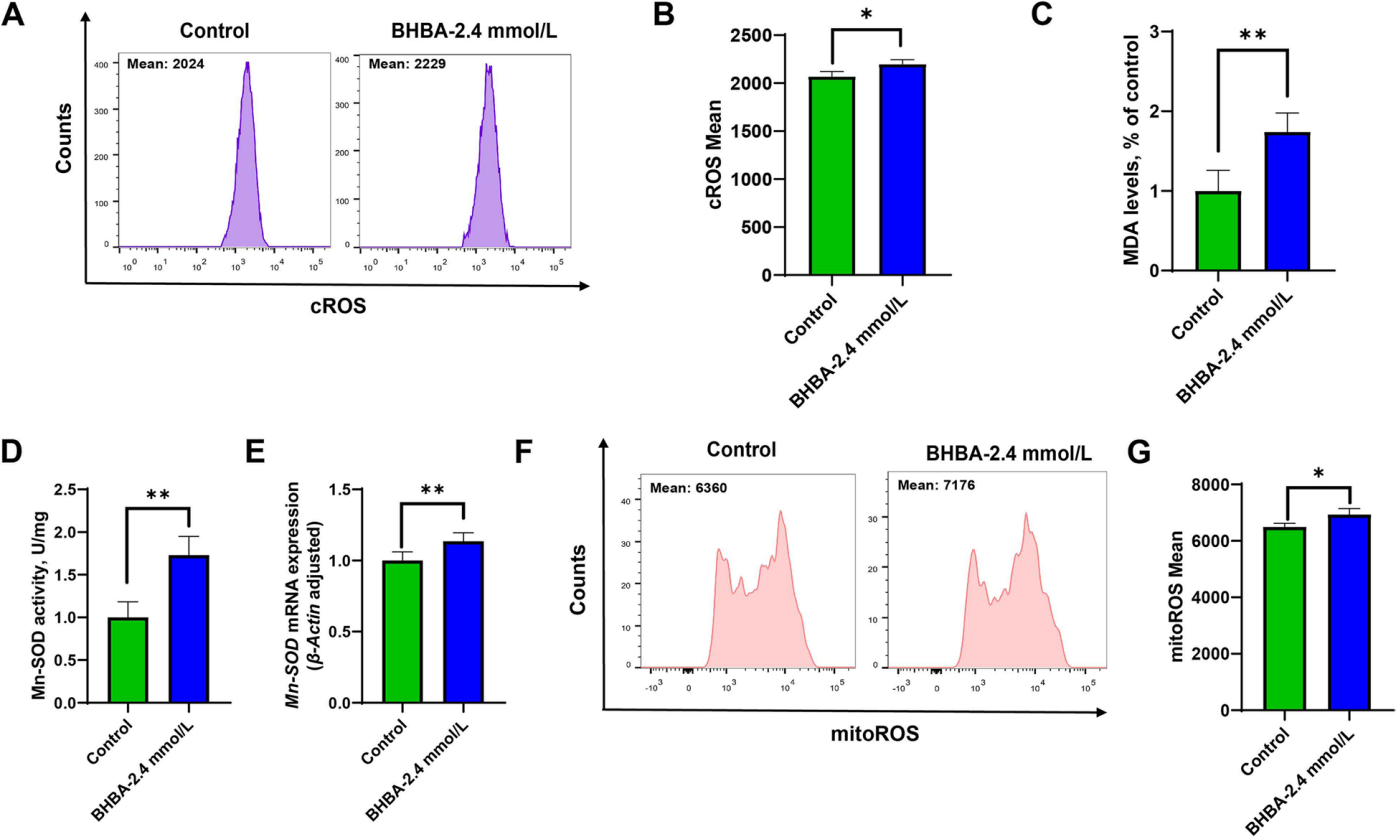
β-Hydroxybutyric acid-induced mitochondrial oxidative stress in fGCs. **A**–**B** cROS levels in the control and BHBA-2.4 mmol/L groups (*n* = 3). (**C**) MDA levels in the control and BHBA-2.4 mmol/L groups (*n* = 6). **D** Mn-SOD activity in the control and BHBA-2.4 mmol/L groups (*n* = 6). **E** *Mn-SOD* mRNA relative expression in the control and BHBA-2.4 mmol/L groups (*n* = 3). **F**–**G** MitoROS levels in the control and BHBA-2.4 mmol/L groups (*n* = 3). For Fig. 2B–E and G, we used Student *t*-tests for significant difference analysis (^*^P < 0.05; ^**^P < 0.01)

Next, we investigate whether the apoptosis of fGCs induced by BHBA was related to peroxidation. Our results showed that cROS and MDA levels were significantly greater in the BHBA-treated group (Fig. 2 A–C). Furthermore, the key mitochondrial antioxidant of Mn-SOD activity and the mRNA expression were significantly increased after 2.4 mmol/L BHBA stimulation (Fig. 2D and E). Consistently, mitoROS levels were also significantly increased after BHBA treatment (Fig. 2 F and G). Our results suggested that the increase in BHBA-induced fGCs apoptosis may be relate to oxidative damage.

### β-Hydroxybutyric acid caused mitochondrial dysfunction in bovine fGCs

**Fig. 3:**
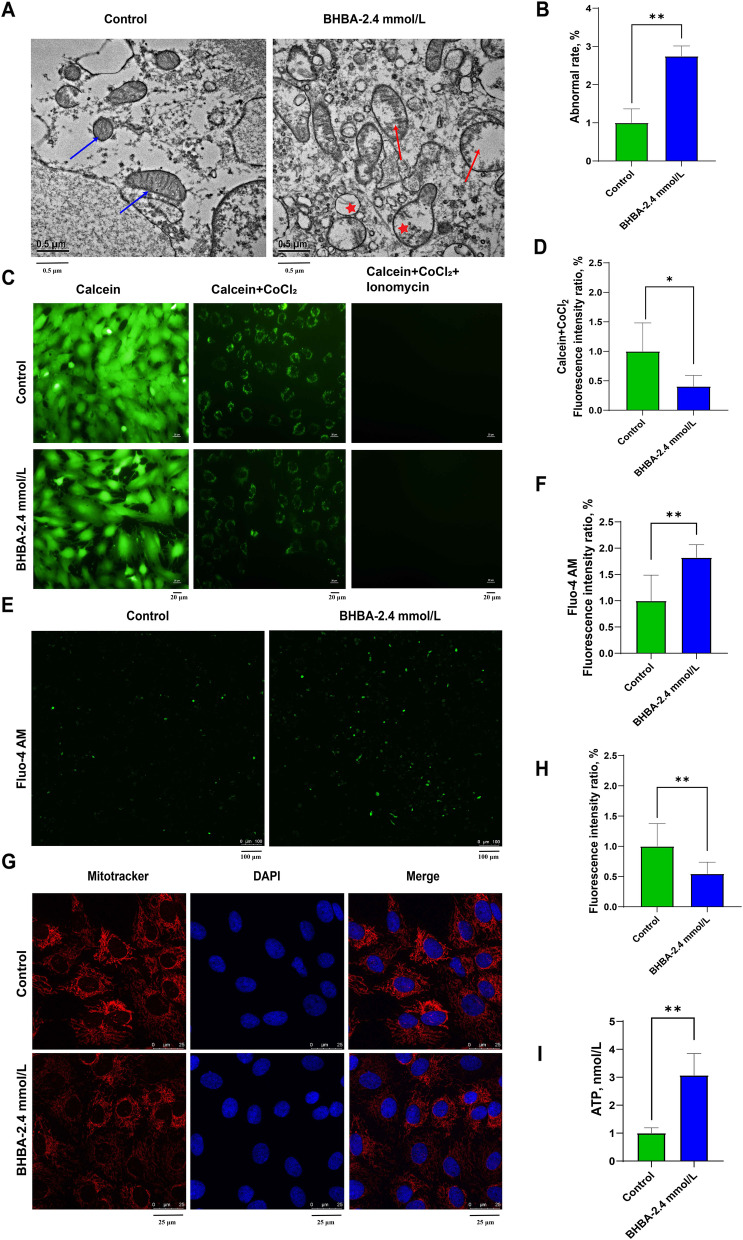
β-Hydroxybutyric acid-induced mitochondrial dysfunction in fGCs. **A** Representative images of the mitochondrial (red arrows) morphology of fGCs after BHBA treatment by TEM (scale bars = 0.5 μm). Normal mitochondria are marked with blue arrows, abnormal mitochondria are marked with red arrows, and autolysosomes are marked with stars. **B** The abnormal rate of mitochondria was statistically analyzed in five images for each treatment group (*n* = 5). **C**–**D** Analysis of MPTP detection and fluorescence intensity after BHBA treatment (scale bars = 20 μm, *n* = 8). Calcein AM showed strong green fluorescence after incubation with cells. After further incubation with CoCl_2_ (Calcein AM + CoCl_2_), the green fluorescence of calcein in the cytoplasm was quenched, and only mitochondria showed green fluorescence, with Ionomycin as a negative control (Calcein AM + CoCl_2_). **E**–**F** Representative images and fluorescence intensity analysis showing intracellular Ca^2+^ levels after BHBA treatment (scale bars = 100 μm, *n* = 5). **G**–**H** Representative images and fluorescence intensity analysis of mitochondrial staining under BHBA stimulation (scale bars = 25 μm, *n* = 8). **I** Bar graph showing intracellular ATP levels after BHBA stimulation (*n* = 3). For Fig. 3B, D, F, H and I, we used Student *t*-tests for significant difference analysis (^*^P < 0.05; ^**^P < 0.01)

We further evaluated mitochondrial architecture and function upon BHBA stimulation. First, we observed a significant increase in abnormal mitochondria (mitochondrial swelling and cristae fragmentation) in fGCs after 2.4 mmol/L BHBA treatment (Fig. 3 A and B). Additionally, 2.4 mmol/L BHBA caused MPTP opening (Fig. 3 C and D) and a significant increase in Ca^2+^ levels (Fig. 3E and F) in fGCs. Similarly, the MitoTracker Red CMXRos staining results showed that BHBA may also reduce mitochondrial mass (Fig. 3G). Since MitoTracker Red CMXRos staining is dependent on MMP, the decreased fluorescence intensity in the BHBA-treated group also implied a decrease in MMP (Fig. 3G and H). Interestingly, ATP levels were significantly greater after BHBA stimulation (Fig. 3I). The increased expression of the mitochondrial fusion gene (*OPA1*) and the decreased expression of the fission gene (*FIS1*) were also consistent with the above results (Fig. S2). These observations indicated that BHBA-induced mitochondrial dysfunction helps to drive injury in fGCs.

### Sirtuin 3 prevented BHBA-induced mitochondrial dysfunction in fGCs

**Fig. 4:**
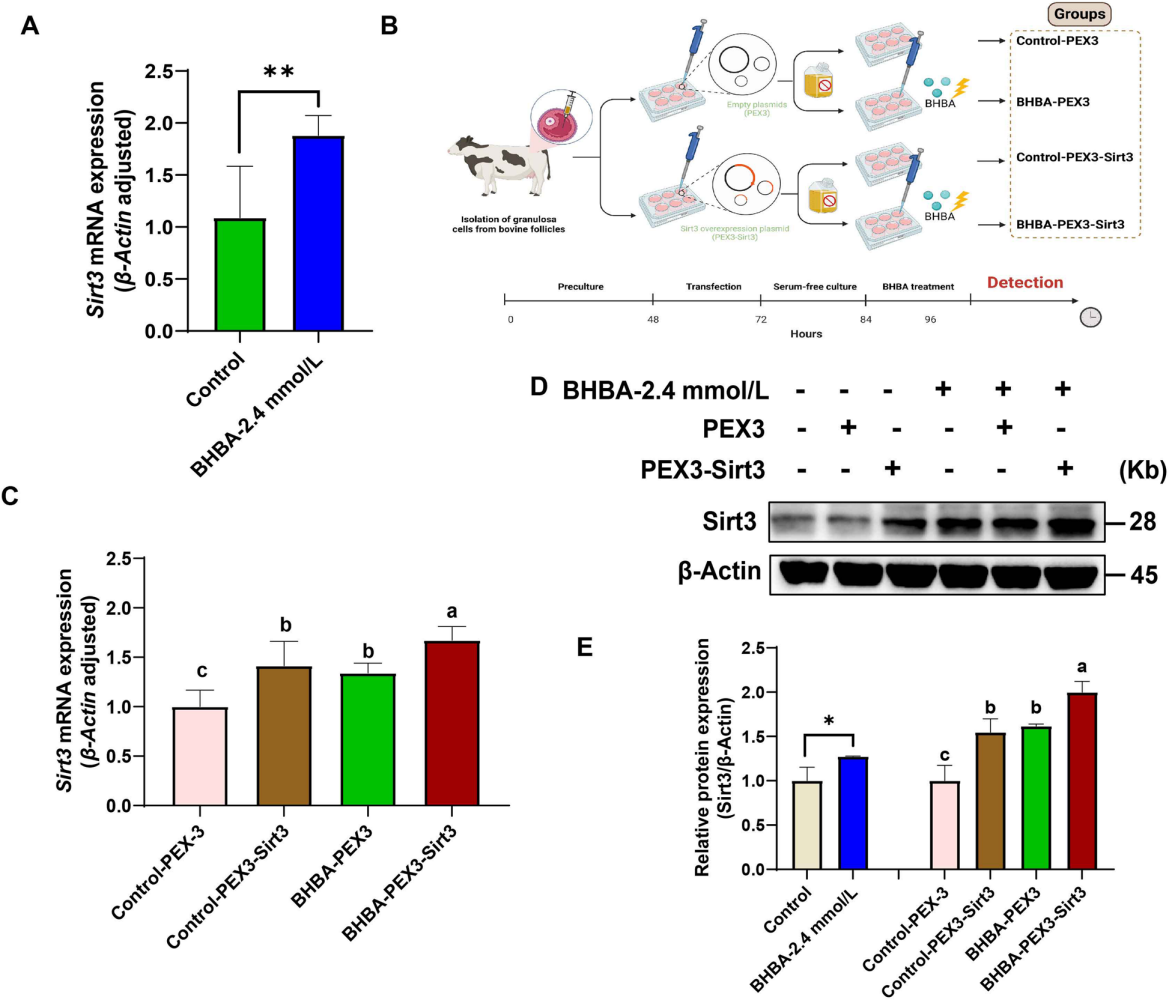
Relative expression of Sirt3 mRNA and protein in different treatment groups. The control group (Control-PEX3) was transfected with PEX3 empty plasmid but not treated with BHBA; the Control-PEX3-*Sirt3* group was transfected with PEX3-*Sirt3* plasmid but not treated with BHBA; the BHBA-PEX3 group was transfected with PEX3 empty plasmid with BHBA treatment; the BHBA-PEX3-*Sirt3* group was transfected with PEX3-*Sirt3* plasmid with BHBA treatment. **A** Sirtuin 3 mRNA relative expression in the control and BHBA-2.4 mmol/L groups (*n* = 3). **B** Schematic protocol for *Sirt3* overexpression with BHBA treatment. **C** The relative expression of *Sirt3* mRNA in different groups following *Sirt3* overexpression with BHBA treatment (*n* = 3). **D**–**E** The relative expression of Sirt3 protein in different groups (*n* = 3). For Fig. 4 A and E (left), we used Student *t*-tests for significant difference analysis (^*^P < 0.05; ^**^P < 0.01); for Fig. 4 C and E (right), we used one-way ANOVA for significant difference analysis (the different lowercases represent *P* < 0.05)

Sirtuin 3 is an important deacetylase localized in mitochondria that regulates mitochondrial function and energy metabolism [39]. First, we found that both the relative expression of mRNA and protein of Sirt3 were significantly increased after 2.4 mmol/L BHBA treatment (Fig. 4 A, D and E). Next, to investigate the regulatory function of Sirt3 in preventing BHBA-induced mitochondrial dysfunction in fGCs, we knocked down or overexpressed *Sirt3*. *Sirtuin 3* was knocked down by using siRNA to observe its regulatory effect on apoptosis of fGCs (Fig. S3). The proliferation rate of fGCs was significantly decreased while the apoptosis rate was significantly increased after 72 h of *Sirt3* knockdown (Fig. S3). Our findings imply that *Sirt3* may positively regulate the development of fGCs. Hence, to probe its protective effect against BHBA-induced injury, we constructed a model of *Sirt3* overexpressing fGCs prior to BHBA stress (Fig. 4B). As expected, the relative expression levels of Sirt3 mRNA and protein were significantly greater in the Control-PEX3-*Sirt3*, BHBA-PEX3 and BHBA-PEX3-*Sirt3* groups (Fig. 4 C, D and E) when compared to the Control-PEX3 group. The successful establishment of the *Sirt3* overexpression-BHBA stress model provided a basis to explore the subsequent regulatory role of *Sirt3* in BHBA-induced mitochondrial dysfunction in fGCs in vitro.

**Fig. 5:**
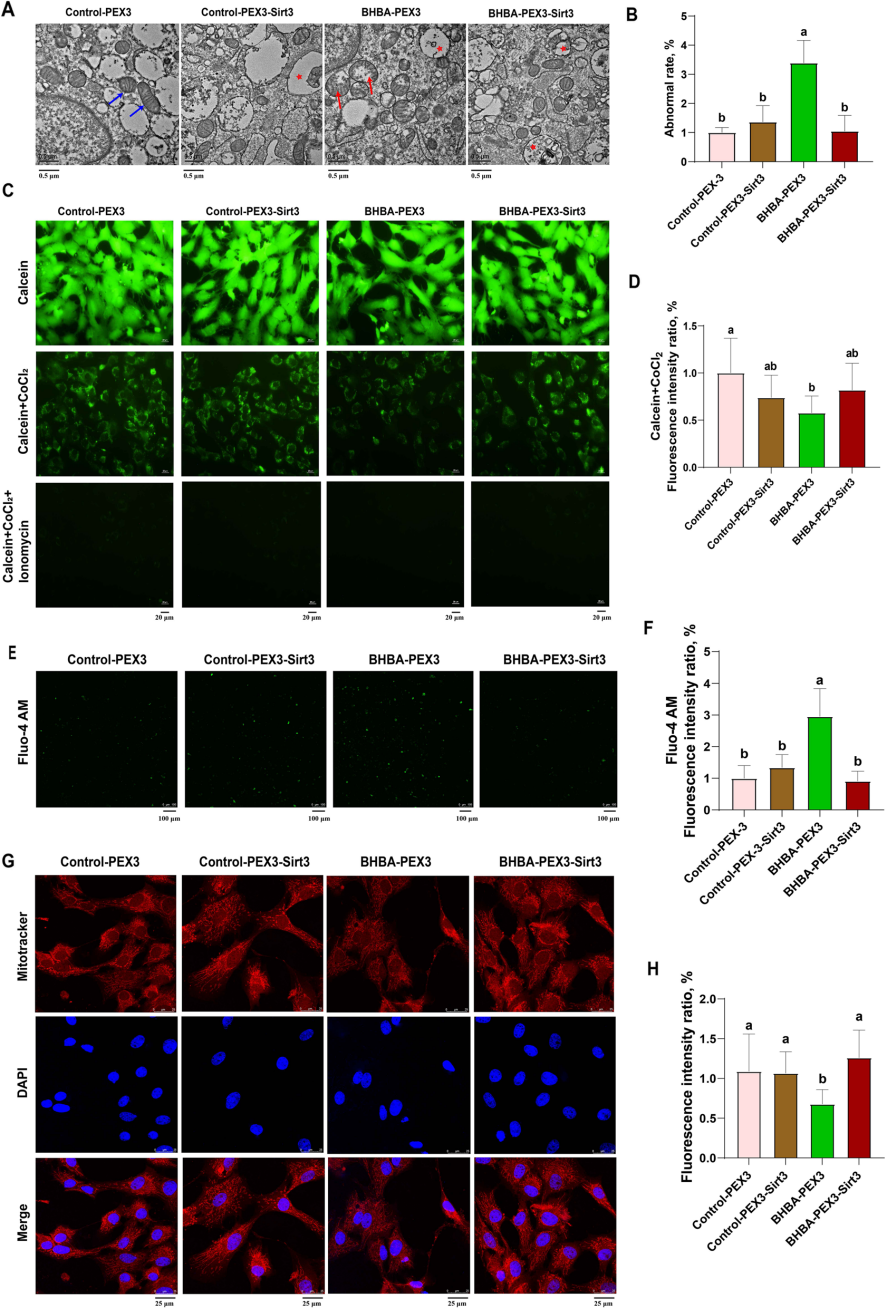
Sirtuin 3 overexpression alleviates BHBA-induced mitochondrial dysfunction in fGCs. **A** Representative images of the mitochondrial morphology of fGCs after the overexpression of *Sirt3* with BHBA treatment by transmission electron microscopy (scale bars = 0.5 μm). Normal form mitochondria are marked with blue arrows, abnormal form mitochondria are marked with red arrows, and autolysosomes are marked with stars. **B** The abnormal rate of mitochondria was statistically analyzed in five images for each treatment group (*n* = 5). **C**–**D** Analysis of MPTP detection and fluorescence intensity after the overexpression of *Sirt3* with BHBA treatment (scale bars = 20 μm, *n* = 8). **E**–**F** Representative images and fluorescence intensity analysis showing intracellular Ca^2+^ levels in different groups (scale bars = 100 μm, *n* = 5). **G**–**H** Representative images and fluorescence intensity analysis of mitochondrial staining in different groups (scale bars = 25 μm, *n* = 8). For Fig. 5B, D, F and H, we used one-way ANOVA for significant difference analysis (the different lowercases represent *P* < 0.05)

We continued to explore whether the overexpression of *Sirt3* could alleviate the mitochondrial dysfunction caused by the induction of BHBA. Consistent with the above findings, the percentage of abnormal mitochondria (Fig. 5 A and B), the degree of MPTP opening (Fig. 5 C and D), Ca^2+^ levels (Fig. E and F) and the MMP (Fig. 5G and H) were significantly increased in the BHBA-PEX3 group; these effects were attenuated by *Sirt3* overexpression pre-treated. Interestingly, there was no significant difference between the Control-PEX3-*Sirt3* and the Control-PEX3 group, implying that *Sirt3* overexpression had little effect on the mitochondrial function of normal fGCs. These results confirm that Sirt3 can prevent BHBA stress-induced mitochondrial dysfunction in fGCs.

### Sirtuin 3 prevented peroxidative damage and apoptosis in bovine fGCs

**Fig. 6:**
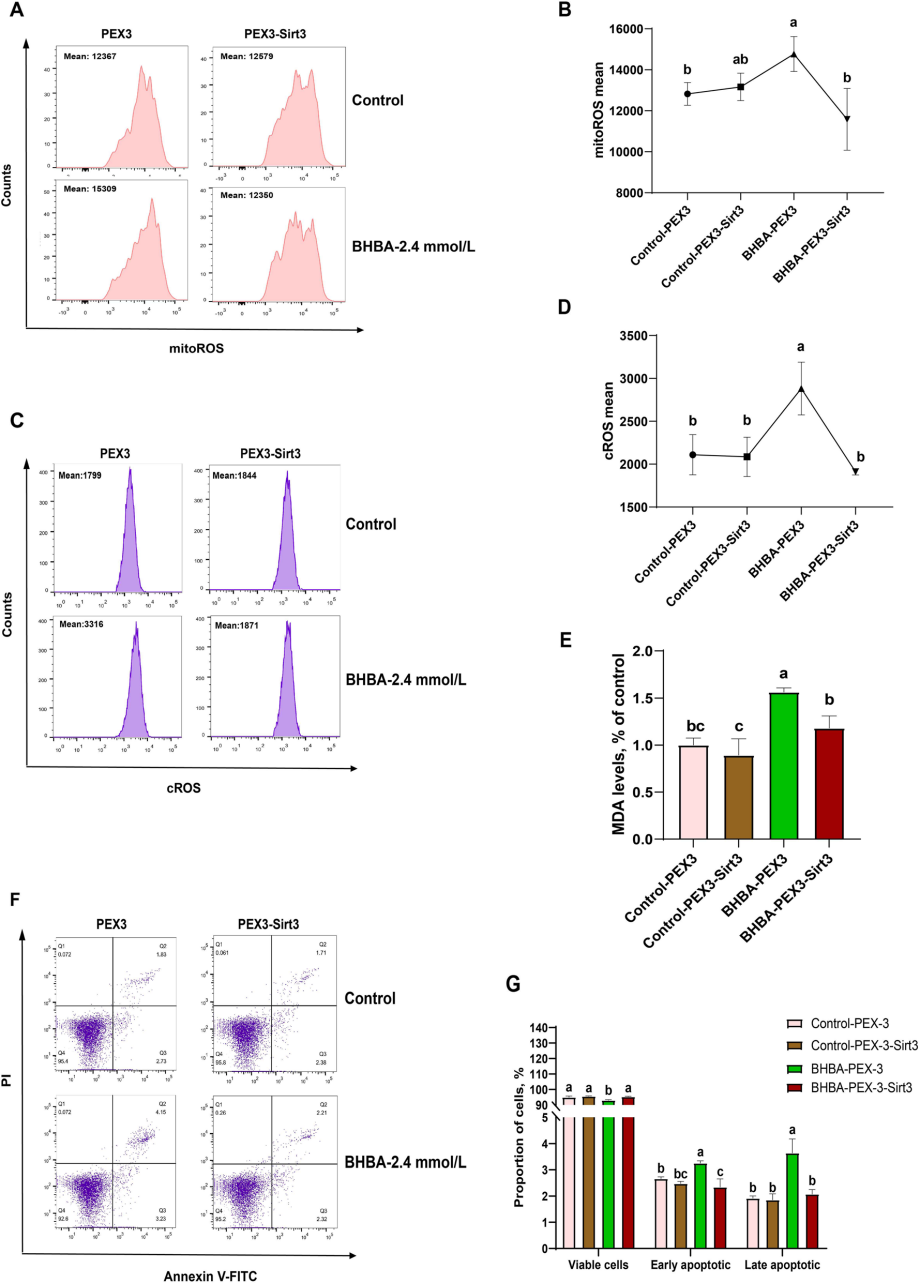
Sirtuin 3 overexpression alleviated BHBA-induced oxidative stress and apoptosis in fGCs. **A**–**B** mitoROS levels analysis in different groups (*n* = 3). **C**–**D** cROS levels analysis in different groups (*n* = 3). **E** MDA levels in different groups (*n* = 6). **F–G** Flow cytometry was used to analyze the apoptosis proportion of fGCs in different treatment groups (*n* = 3). The bar graph shows the apoptosis proportion. For Fig. 6B, D, E and G, we used one-way ANOVA for significant difference analysis (the different lowercases represent *P* < 0.05)

To provide further evidence of the protective effect of Sirt3, we examined oxidative stress and apoptosis in the *Sirt3* overexpression-BHBA stress model. As shown in Fig. 6 A to D, mitoROS and cROS levels were significantly increased in the BHBA-PEX3 group, as were MDA levels (Fig. 6E). However, the overexpression of *Sirt3* alleviated the upregulation of ROS and MDA in fGCs. Similarly, the proportion of viable cells in the BHBA-PEX3 group were significantly decreased while the proportion of early apoptotic and late apoptotic proportion was significantly increased (Fig. 6 F–G). As expected, the overexpression of *Sirt3* pretreatment reduced the BHBA induction of apoptosis in fGCs. Overall, our results demonstrate that Sirt3 prevents BHBA-triggered oxidative stress and apoptosis.

### Involvement of the AMPK-mTOR pathway in Sirt3-induced protection

**Fig. 7:**
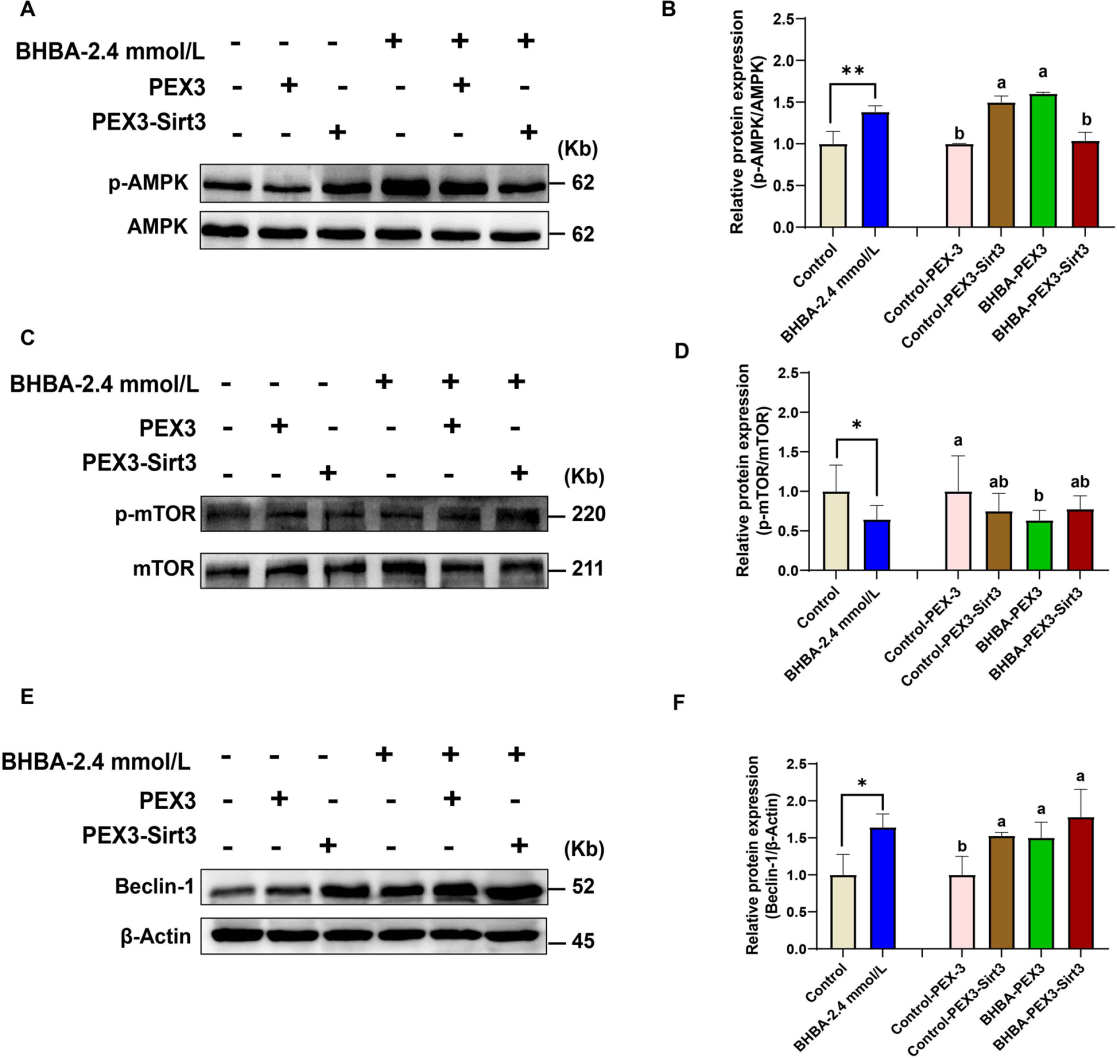
The overexpression of *Sirt3* may alleviate BHBA-induced injury by enhancing autophagy by the regulation of the AMPK-mTOR pathway. The p-AMPK (**A**–**B**), p-mTOR (**C**–**D**), and the relative expression of Beclin-1 (**E–F**) in different groups as detected by western blotting (*n* = 3). For the left of Fig. 7B, D and F, we used Student *t*-tests for significant difference analysis (^*^P < 0.05; ^**^P < 0.01); for the right of Fig. 7B, D and F, we used one-way ANOVA for significant difference analysis (the different lowercases represent *P* < 0.05)

Next, we considered how Sirt3 protects fGCs from BHBA-induced damage. Previous studies have demonstrated the key role of the AMPK-mTOR pathway in Sirt3-induced protection mechanisms [23, 40–42]. To verify the effectiveness of the AMPK-mTOR pathway in this study, we first detected the phosphorylation of AMPK (p-AMPK) and mTOR (p-mTOR) after BHBA stress by western blotting. As shown in Fig. 7 A and B, the relative expression of p-AMPK protein increased significantly after BHBA stimulation; similar findings were evident in the group overexpressing Sirt3 without BHBA treatment. However, when BHBA treatment was followed by the overexpression of Sirt3, the expression levels of p-AMPK decreased. In contrast, the expression of p-mTOR significantly decreased after BHBA stimulation, whereas BHBA stimulation after overexpression of Sirt3 pretreatment resulted in an increase in p-mTOR (Fig. 7 C and D). Therefore, the AMPK-mTOR signaling pathway may be involved in the protection of Sirt3 against fGCs.

The Sirt3-AMPK-mTOR pathway has been shown to regulate mitochondrial autophagy and removes impaired organelles in cells and plays a pivotal role in maintaining intracellular homeostasis [36]. To further elucidate the pathway by which Sirt3 protects fGCs after BHBA stimulation, we investigated whether activation of AMPK-mTOR contributes to mitochondrial autophagy. Accordingly, we further explored the expression of the mitochondrial autophagy-related proteins Beclin-1. Our data showed that the expression of Beclin-1 was significantly increased in the BHBA-treated group; interestingly, *Sirt3* overexpression enhanced the expression of Beclin-1 further (Fig. 7E and F). These results implied that BHBA-triggered mitochondrial disorder could trigger mitochondrial autophagy in fGCs. Furthermore, *Sirt3* overexpression can protect fGCs from the injury caused by BHBA stimulation by enhancing autophagy function and reducing mitochondrial dysfunction.

## Discussion

The estrous cycle occurs following follicular development and steroid hormone synthesis; in particular, fGCs are decisive in regulating follicular development and hormone synthesis activities [43–46]. Increased BHBA in the blood of ketotic cows lead to the elevated generation of deleterious lipids, impaired cellular function and oxidative stress-related disease. In particular, oxidative stress is a major cause of female infertility because the accumulation of intracellular ROS caused by oxidative stress leads to oxidative damage of fGCs, which in turn triggers follicular atresia and ovulation disorders. Sirtuin 3 is a mitochondrial deacetylase that plays a critical role in regulating metabolic and antioxidant functions [29, 47, 48]. Here, we provide direct evidence that Sirt3 ultimately contributes to alleviating BHBA-induced oxidative stress and mitochondrial dysfunction in fGCs by promoting autophagy followed with the AMPK-mTOR-Beclin-1 pathway activated.

The fGCs are instrumental in determining follicular fate and coordinate the follicular development cycle through apoptosis-induced follicular atresia [13, 49, 50]. However, the excessive apoptosis of fGCs can cause abnormal follicular atresia, thus affecting follicular utilization and reducing fertility [51]. Herein, we detected an increase in the apoptotic rate of fGCs as well as pro-apoptotic-related proteins with increasing BHBA concentrations. Similarly, in our previous study, we identified that the differentially expressed genes were involved in apoptosis pathway after 2.4 mmol/L BHBA stress [52]. This suggests that increased concentrations of BHBA-induced apoptosis in fGCs; this may be an impaired developmental factor of follicles. Consistently, excessive BHBA stress has been shown to reduce the activity of multiple types of cells [7, 53, 54]. Notably, a recent study proved that follicle growth rate was reduced after BHBA injection for 72 h [12]. Yet, BHBA treatment for 6 h did not alter the steroid concentration in FF or the abundance of mRNA transcripts involved in regulating important functions of preovulatory of fGCs [12]. These investigators proposed a possible reason for these findings in that the experimental cows were not in NEB and the BHBA may have been used up by the cells, thus resulting in a reduction in follicular BHBA concentration and the restoration of fGCs function and ovulation [12]. In addition, the BHBA treatment time should also not be neglected; the longer time period of 12 h for BHBA stress used in our study may also be the reason for such discrepancy. This may suggest that BHBA plays a dual role, as it can be used as an energetic substance for cellular use, while an excess of BHBA may also cause oxidative stress resulting in cellular injury; this is closely associated with the state of cellular energy (such as by starvation).

Mitochondrial dysfunction could activate the mitochondrial apoptotic pathway leading to apoptosis and has been considered as a key driver of cell injury and apoptosis [19, 55]. Therefore, we further explored the mechanism by which BHBA promotes apoptosis in fGCs by focusing on mitochondrial dysfunction. Mitochondria play a vital role in maintaining energy metabolism and redox homeostasis as a major regulator of ATP and ROS production [56]. Previous researchers proposed a positive feedback effect of “ROS-induced ROS release” for the sequential relationship between oxidative stress and mitochondrial dysfunction: increased oxidative stress damages mitochondria thus leading to mitochondrial dysfunction and the subsequent production of excess mitoROS, thus causing cellular damage [57, 58]. Similarly, we found that BHBA stress induced an increase in mitoROS and activated the antioxidant system in fGCs. An increase in mitochondrial Mn-SOD could reduce mitoROS and prevent cellular damage [59]. In our study, both mitoROS and Mn-SOD were increased after BHBA stress, thus indicating that the fGCs rely on the antioxidant system to actively fight mitoROS. Previous studies have demonstrated that BHBA induces oxidative stress in a variety of bovine cells, including endometrial cells [7], abomasum smooth muscle cells [60] and hepatocytes [61]; these findings were consistent with our current data.

In addition, we further found that BHBA stimulation increased the proportion of abnormal mitochondrial morphology (including mitochondrial swelling and cristae fragmentation) in fGCs. The main cause of mitochondrial swelling may be due to the opening of the MPTP; we consistently observed that BHBA triggered the opening of the MPTP. Previous research showed that MPTP opening is also an early sign of mitochondrial oxidative stress induced by cellular damage [62–64]. Mitochondrial oxidative stress can induce a reduction of MMP and the release of Ca^2+^, thus activating the subsequent caspase cascade reaction [57, 65–67]. In the present study, 2.4 mmol/L BHBA caused a decrease in MMP and Ca^2+^ release in fGCs, thus indicating that BHBA-induced mitochondrial oxidative stress and mitochondrial dysfunction may be responsible for apoptosis in fGCs. Surprisingly, even though BHBA damaged mitochondria, leading to dysfunction, we found that the ATP content was increased; this may be related to increased mitochondrial fusion. We acknowledge the view that activation of the apoptotic system can uncouple the mitochondrial electron transport chain and down-regulate the level of ATP production [68]. Yet, the observed increase in ATP may be related to the properties of BHBA as an energetic substance; however, this requires further investigation.

Sirtuin 3 is a deacetylase predominantly found in the mitochondria that plays an increasingly essential role in maintaining homeostasis of mitoROS by the deacetylation of substrates involved in ROS production or detoxification [23, 69, 70]. Intriguingly, the relative expression of Sirt3 was increased in fGCs when exposed to high concentrations of BHBA. Therefore, we hypothesized that under BHBA-overload, Sirt3 may serve as a feedback mechanism to balance redox homeostasis. To prove our point, we overexpressed *Sirt3* in fGCs before exposure to BHBA stress. We observed that Sirt3 overexpression significantly improve mitochondrial morphology and functions, while reducing the cROS and mitoROS production and alleviating oxidative stress. Consistently, a previous study showed that Sirt3 overexpression could reduce the ROS increase in oocytes caused by a high fat diet [71]. Furthermore, Sirt3 was found to sense and regulate ROS production to play an active role in folliculogenesis and luteinization in GCs [72]; this concurred with our present findings. Recent study has shown that Sirt3 is crucial for autophagy activation, which reduces oxidative damage and ROS levels by removing damaged organelles (e.g., mitochondria) [73]. Our findings showed enhanced autophagic signaling pathway after overexpression of Sirt3, which may be an essential factor for Sirt3 to alleviate BHBA-induced oxidative stress and mitochondrial disorders.

Mechanistically, there is a positive feedback loop between Sirt3 and AMPK: Sirt3 deacetylates and activates LKB1 (liver kinase) activity which phosphorylates AMPK and activates AMPK [23, 74, 75]. Previous data also showed that Sirt3 may regulate FAO by deacetylating LKB1 and activating AMPK; the activation of Sirt3 reduced ROS and lipid peroxidation by improving mitochondrial function [76]. In this study, both Sirt3 overexpression and BHBA stimulation increased the expression of p-AMPK; interestingly, p-AMPK decreased after *Sirt3* overexpression following BHBA stimulation. The increased Sirt3 may regulate mitochondrial function in fGCs by activating p-AMPK through the deacetylation of LKB1; once subjected to BHBA stress, AMPK activates downstream molecules to protect fGCs from restoring function [77]. This may explain the decreased level of p-AMPK after the overexpression of Sirt3 followed by BHBA treatment. In addition, the AMPK pathway activates autophagy by phosphorylating S317 and S777 to inhibit mTOR [78, 79]. Emerging evidence shows that Sirt3 could regulate mitochondrial autophagy and protect cells from oxidative stress-induced damage by activating the AMPK-mTOR pathway [40, 41, 80, 81]. As expected, in contrast to p-AMPK expression levels, increased BHBA decreased p-mTOR, whereas *Sirt3* overexpression followed by BHBA stimulation of p-mTOR attenuated this decrease. Here, we also provide an important clue that the expression of Beclin-1 increases after BHBA stimulation. Furthermore, the overexpression of Sirt3 before BHBA stress enhanced the expression of Beclin-1 even more and more autophagic vesicles were observed. These results indicated that Sirt3 exerted a positive influence on autophagy. Therefore, the AMPK-mTOR-Beclin-1 pathway may be involved in the protective mechanism of Sirt3-regulated autophagy against mitochondrial damage by BHBA stress.

## Conclusion

**Fig. 8:**
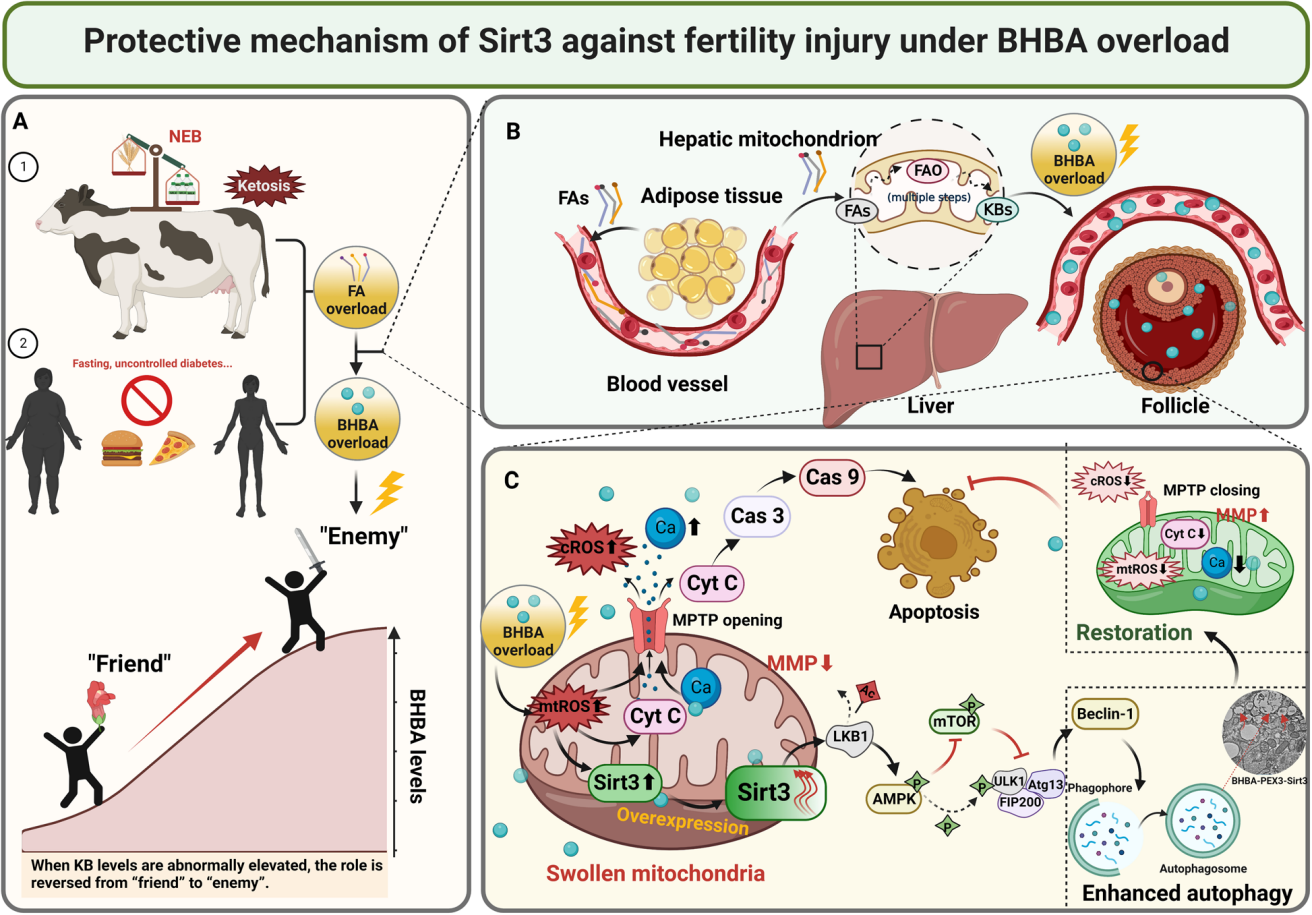
The possible mechanism by which Sirt3 protected fGCs from BHBA-induced cell injury. Sirtuin 3 prevented BHBA-induced oxidative stress and mitochondrial dysfunction through the AMPK-mTOR-Beclin-1 signaling pathway which may be associated with autophagy

In conclusion, stimulation with high concentrations of BHBA can cause mitochondrial oxidative stress and mitochondrial dysfunction, ultimately triggering apoptosis in fGCs. In addition, Sirt3 regulates autophagy in fGCs through the AMPK-mTOR-Beclin-1 pathway to alleviate BHBA-induced injury and reduce the rate of apoptosis (Fig. 8). This study elucidates the mechanism of injury to bovine fGCs by elevated BHBA concentration, while providing a reference for clarifying the prevention of mechanisms underlying fertility disorders caused by ketosis in dairy cows.

## Supplementary Information

**Additional file 1: Table S1.** Antibody information.

**Additional file 2: Table S2.** Nucleotide information.

**Additional file 3: Fig. S1.** The specific sequences of the Sirt3 overexpression plasmid.

**Additional file 4: Fig. S2.** Relative expression analysis of mitochondrial fusion (OPA1) and division(FIS1)-related genes. *P < 0.05; **P < 0.01.

**Additional file 5: Fig. S3.** The effect of *Sirt3* knockdown on the proliferation and apoptosis of fGCs. (A) Assay showing siRNA interference efficiency (B) Analysis of theproliferation rate of fGCs after *Sirt3* knockdown for 24 h, 48 and72 h. (C-D) Analysis of the apoptosis rate of fGCs after *Sirt3* knockdown for72 h. ^*^P < 0.05; ^**^P < 0.01.

## Data Availability

The datasets used and/or analysed during the current study are available from the corresponding author on reasonable request.

## Abbreviations

BHBA: β-Hydroxybutyric acid
cROS: Cytosolic reactive oxygen species
FAO: Fatty acid oxidation
FBS: Fetal bovine serum
FF: Follicular fluid
fGCs: Follicular granulosa cells
FSHR: Follicle stimulating hormone receptor
KBs: Ketone bodies
MDA: Malondialdehyde
mitoROS: Mitochondria reactive oxygen species
MMP: Mitochondrial membrane potential
MPTP: Mitochondrial permeability transition pore
NEB: Negative energy balance
OD: Optical density
qRT-PCR: Quantitative real-time polymerase chain reaction
ROS: Reactive oxygen species
SD: Standard deviation
SDS-PAGE: SDS-polyacrylamide gel electrophoresis
Sirt3: Sirtuin 3
TEM: Transmission electron microscopy

## References

[1] Rojas-Morales P, Pedraza-Chaverri J, Tapia E (2020). Ketone bodies, stress response, and redox homeostasis. Redox Biol.

[2] Puchalska P, Crawford PA (2021). Metabolic and signaling roles of ketone bodies in health and disease. Annu Rev Nutr.

[3] Kanikarla-Marie P, Jain SK (2016). Hyperketonemia and ketosis increase the risk of complications in type 1 diabetes. Free Radic Biol Med.

[4] Sangalli JR, Sampaio RV, Del Collado M, da Silveira JC, De Bem THC, Perecin F (2018). Metabolic gene expression and epigenetic effects of the ketone body β-hydroxybutyrate on H3K9ac in bovine cells, oocytes and embryos. Sci Rep.

[5] Shen T, Xu F, Fang Z, Loor JJ, Ouyang H, Chen M (2021). Hepatic autophagy and mitophagy status in dairy cows with subclinical and clinical ketosis. J Dairy Sci.

[6] Rutherford AJ, Oikonomou G, Smith RF (2016). The effect of subclinical ketosis on activity at estrus and reproductive performance in dairy cattle. J Dairy Sci.

[7] Cheng X, Yang S, Xu C, Li L, Zhang Y, Guo Y (2019). Proanthocyanidins protect against β-Hydroxybutyrate-induced oxidative damage in bovine endometrial cells. Molecules.

[8] Leroy J (2004). Metabolic changes in follicular fluid of the dominant follicle in high-yielding dairy cows early post partum. Theriogenology.

[9] Hostens M, Fievez V, Vlaeminck B, Buyse J, Leroy J, Piepers S (2011). The effect of marine algae in the ration of high-yielding dairy cows during transition on metabolic parameters in serum and follicular fluid around parturition. J Dairy Sci.

[10] Wang L, Chen P, Xiao W (2021). β-hydroxybutyrate as an anti-aging metabolite. Nutrients.

[11] Li Y, Ding HY, Wang XC, Feng SB, Li XB, Wang Z (2016). An association between the level of oxidative stress and the concentrations of NEFA and BHBA in the plasma of ketotic dairy cows. J Anim Physiol Anim Nutr (Berl).

[12] Missio D, Fritzen A, Cupper Vieira C, Germano Ferst J, Farias Fiorenza M, Guedes de Andrade L (2022). Increased β-hydroxybutyrate (BHBA) concentration affect follicular growth in cattle. Anim Reprod Sci.

[13] Matsuda F, Inoue N, Manabe N, Ohkura S (2012). Follicular growth and atresia in mammalian ovaries: regulation by survival and death of granulosa cells. J Reprod Dev.

[14] Yang F, Pei R, Zhang Z, Liao J, Yu W, Qiao N (2019). Copper induces oxidative stress and apoptosis through mitochondria-mediated pathway in chicken hepatocytes. Toxicol In Vitro.

[15] Shen M, Cao Y, Jiang Y, Wei Y, Liu H (2018). Melatonin protects mouse granulosa cells against oxidative damage by inhibiting FOXO1-mediated autophagy: implication of an antioxidation-independent mechanism. Redox Biol.

[16] Wang Y, Yang C, Elsheikh NAH, Li C, Yang F, Wang G (2019). HO-1 reduces heat stress-induced apoptosis in bovine granulosa cells by suppressing oxidative stress. Aging.

[17] Park SA, Joo NR, Park JH, Oh SM (2021). Role of the SIRT1/p53 regulatory axis in oxidative stress–mediated granulosa cell apoptosis. Mol Med Rep.

[18] Zhang M, Zhang Q, Hu Y, Xu L, Jiang Y, Zhang C (2017). miR-181a increases FoxO1 acetylation and promotes granulosa cell apoptosis via SIRT1 downregulation. Cell Death Dis.

[19] Sun Y, Ge X, Li X, He J, Wei X, Du J (2020). High-fat diet promotes renal injury by inducing oxidative stress and mitochondrial dysfunction. Cell Death Dis.

[20] Sharaf MS, Stevens D, Kamunde C (2017). Zinc and calcium alter the relationship between mitochondrial respiration, ROS and membrane potential in rainbow trout (Oncorhynchus mykiss) liver mitochondria. Aquat Toxicol.

[21] Kang P, Zhang W, Chen X, Yi X, Song P, Chang Y (2018). TRPM2 mediates mitochondria-dependent apoptosis of melanocytes under oxidative stress. Free Radic Biol Med.

[22] Wang T, Cao Y, Zheng Q, Tu J, Zhou W, He J (2019). SENP1-Sirt3 signaling controls mitochondrial protein acetylation and metabolism. Mol cell.

[23] Liu S, Su Y, Sun B, Hao R, Pan S, Gao X (2020). Luteolin protects against CIRI, potentially via regulation of the SIRT3/AMPK/mTOR signaling pathway. Neurochem Res.

[24] Yi X, Guo W, Shi Q, Yang Y, Zhang W, Chen X (2019). SIRT3-dependent mitochondrial dynamics remodeling contributes to oxidative stress-induced melanocyte degeneration in vitiligo. Theranostics.

[25] Wang Z, Sun R, Wang G, Chen Z, Li Y, Zhao Y (2020). SIRT3-mediated deacetylation of PRDX3 alleviates mitochondrial oxidative damage and apoptosis induced by intestinal ischemia/reperfusion injury. Redox Biol.

[26] Zheng J, Shi L, Liang F, Xu W, Li T, Gao L (2018). Sirt3 ameliorates oxidative stress and mitochondrial dysfunction after intracerebral hemorrhage in diabetic rats. Front Neurosci.

[27] Gao J, Feng Z, Wang X, Zeng M, Liu J, Han S (2018). SIRT3/SOD2 maintains osteoblast differentiation and bone formation by regulating mitochondrial stress. Cell Death Differ.

[28] Zhu Z, Li H, Chen W, Cui Y, Huang A, Qi X. Perindopril improves cardiac function by enhancing the expression of SIRT3 and PGC-1α in a rat model of isoproterenol-induced cardiomyopathy. Front Pharmacol. 2020;11:94. 10.3389/fphar.2020.00094. 10.3389/fphar.2020.00094 PMC7046591 32153406

[29] Dikalova AE, Pandey A, Xiao L, Arslanbaeva L, Sidorova T, Lopez MG (2020). Mitochondrial deacetylase Sirt3 reduces vascular dysfunction and hypertension while Sirt3 depletion in essential hypertension is linked to vascular inflammation and oxidative stress. Circ Res.

[30] Wang Q, Xu J, Li X, Liu Z, Han Y, Xu X (2019). Sirt3 modulate renal ischemia-reperfusion injury through enhancing mitochondrial fusion and activating the ERK-OPA1 signaling pathway. J Cell Physiol.

[31] Liu J, Yan W, Zhao X, Jia Q, Wang J, Zhang H (2019). Sirt3 attenuates post-infarction cardiac injury via inhibiting mitochondrial fission and normalization of AMPK-Drp1 pathways. Cell Signal.

[32] Eid RA, Bin-Meferij MM, El-Kott AF, Eleawa SM, Zaki MSA, Al-Shraim M (2021). Exendin-4 protects against myocardial ischemia-reperfusion injury by upregulation of SIRT1 and SIRT3 and activation of AMPK. J Cardiovasc Transl Res.

[33] Li Y, Hu K, Liang M, Yan Q, Huang M, Jin L (2021). Stilbene glycoside upregulates SIRT3/AMPK to promotes neuronal mitochondrial autophagy and inhibit apoptosis in ischemic stroke. Adv Clin Exp Med.

[34] Xin T, Lu C (2020). Sirt3 activates AMPK-related mitochondrial biogenesis and ameliorates sepsis-induced myocardial injury. Aging.

[35] Zhang JJ, Li YQ, Wang YS, Chen L, Wang XZ (2021). Estradiol ameliorates metformin-inhibited sertoli cell proliferation via AMPK/TSC2/mTOR signaling pathway. Theriogenology.

[36] Hwang HY, Shim JS, Kim D, Kwon HJ (2021). Antidepressant drug sertraline modulates AMPK-MTOR signaling-mediated autophagy via targeting mitochondrial VDAC1 protein. Autophagy.

[37] Herzig S, Shaw RJ (2018). AMPK: guardian of metabolism and mitochondrial homeostasis. Nat Rev Mol Cell Biol.

[38] Zhang H, Luo Q, Lu X, Yin N, Zhou D, Zhang L (2018). Effects of hPMSCs on granulosa cell apoptosis and AMH expression and their role in the restoration of ovary function in premature ovarian failure mice. Stem Cell Res Ther.

[39] Tomczyk MM, Cheung KG, Xiang B, Tamanna N, Fonseca Teixeira AL, Agarwal P (2022). Mitochondrial sirtuin-3 (SIRT3) prevents doxorubicin-induced dilated cardiomyopathy by modulating protein acetylation and oxidative stress. Circ Heart Fail.

[40] Dai SH, Chen T, Li X, Yue KY, Luo P, Yang LK (2017). Sirt3 confers protection against neuronal ischemia by inducing autophagy: involvement of the AMPK-mTOR pathway. Free Radic Biol Med.

[41] Han D, Jiang L, Gu X, Huang S, Pang J, Wu Y (2020). SIRT3 deficiency is resistant to autophagy-dependent ferroptosis by inhibiting the AMPK/mTOR pathway and promoting GPX4 levels. J Cell Physiol.

[42] Zhao W, Zhang L, Chen R, Lu H, Sui M, Zhu Y (2018). SIRT3 protects against acute kidney injury via AMPK/mTOR-regulated autophagy. Front Physiol.

[43] Shi J, Liu C, Chen M, Yan J, Wang C, Zuo Z (2021). The interference effects of bisphenol A on the synthesis of steroid hormones in human ovarian granulosa cells. Environ Toxicol.

[44] Minegishi T, Tsuchiya M, Hirakawa T, Abe K, Inoue K, Mizutani T (2000). Expression of steroidogenic acute regulatory protein (StAR) in rat granulosa cells. Life Sci.

[45] Scully S, Evans ACO, Duffy P, Crowe MA (2014). Characterization of follicle and CL development in beef heifers using high resolution three-dimensional ultrasonography. Theriogenology.

[46] De Rensis F, Kirkwood RN (2016). Control of estrus and ovulation: fertility to timed insemination of gilts and sows. Theriogenology.

[47] Tao R, Vassilopoulos A, Parisiadou L, Yan Y, Gius D (2014). Regulation of MnSOD enzymatic activity by Sirt3 connects the mitochondrial acetylome signaling networks to aging and carcinogenesis. Antioxid Redox Signal.

[48] Hirschey MD, Shimazu T, Goetzman E, Jing E, Schwer B, Lombard DB (2010). SIRT3 regulates mitochondrial fatty-acid oxidation by reversible enzyme deacetylation. Nature.

[49] Zhang J, Xu Y, Liu H, Pan Z (2019). MicroRNAs in ovarian follicular atresia and granulosa cell apoptosis. Reprod Biol Endocrinol.

[50] Li X, Lin Y, Yao J, Pan B, Zhan X, Chen Z (2022). Protegrin-1 inhibits porcine ovarian granulosa cell apoptosis from H(2)O(2)-induced oxidative stress via the PERK/eIF2α/CHOP signaling pathway in vitro. Theriogenology.

[51] Zhang X, Yu T, Guo X, Zhang R, Jia Y, Shang C (2021). Ufmylation regulates granulosa cell apoptosis via ER stress but not oxidative stress during goat follicular atresia. Theriogenology.

[52] Gong J, Zhao S, Heng N, Wang Y, Hu Z, Wang H, et al. The dynamic transcription profiles of proliferating bovine ovarian granulosa when exposed to increased levels of β-hydroxybutyric acid. Front Vet Sci. 2022;9:915956. 10.3389/fvets.2022.915956. 10.3389/fvets.2022.915956 PMC9389329 35990259

[53] Zou D, Liu R, Shi S, Du J, Tian M, Wang X (2021). BHBA regulates the expressions of lipid synthesis and oxidation genes in sheep hepatocytes through the AMPK pathway. Res Vet Sci.

[54] Li P, Li L, Zhang C, Cheng X, Zhang Y, Guo Y, et al. Palmitic acid and β-hydroxybutyrate induce inflammatory responses in bovine endometrial cells by activating oxidative stress-mediated NF-κB signaling. Molecules. 2019;24(13):2421. 10.3390/molecules24132421. 10.3390/molecules24132421 PMC6650895 31266188

[55] Zhan M, Brooks C, Liu F, Sun L, Dong Z (2013). Mitochondrial dynamics: regulatory mechanisms and emerging role in renal pathophysiology. Kidney Int.

[56] Ramzan R, Dolga AM, Michels S, Weber P, Culmsee C, Rastan AJ (2022). Cytochrome c oxidase inhibition by ATP decreases mitochondrial ROS production. Cells.

[57] Kim SH, Kim H (2018). Inhibitory effect of astaxanthin on oxidative stress-induced mitochondrial dysfunction. Nutrients.

[58] Zorov DB, Filburn CR, Klotz LO, Zweier JL, Sollott SJ (2000). Reactive oxygen species (ROS)-induced ROS release: a new phenomenon accompanying induction of the mitochondrial permeability transition in cardiac myocytes. J Exp Med.

[59] Sinha K, Das J, Pal PB, Sil PC (2013). Oxidative stress: the mitochondria-dependent and mitochondria-independent pathways of apoptosis. Arch Toxicol.

[60] Tian W, Wei T, Li B, Wang Z, Zhang N, Xie G (2014). Pathway of programmed cell death and oxidative stress induced by β-hydroxybutyrate in dairy cow abomasum smooth muscle cells and in mouse gastric smooth muscle. PLoS ONE.

[61] Song Y, Li N, Gu J, Fu S, Peng Z, Zhao C (2016). β-Hydroxybutyrate induces bovine hepatocyte apoptosis via an ROS-p38 signaling pathway. J Dairy Sci.

[62] Kaasik A, Safiulina D, Zharkovsky A, Veksler V (2007). Regulation of mitochondrial matrix volume. Am J Physiol Cell Physiol.

[63] Jang S, Javadov S (2020). OPA1 regulates respiratory supercomplexes assembly: the role of mitochondrial swelling. Mitochondrion.

[64] Zhao Q, Liu J, Deng H, Ma R, Su S (2021). Targeting mitochondria-located circRNA SCAR alleviates NASH via reducing mROS output. Cell.

[65] Burke PJ, Mitochondria (2017). Bioenergetics and apoptosis in cancer. trends cancer.

[66] Pan K, Zhang W, Chen X, Yi X, Pu S, Chang Y (2018). TRPM2 mediates mitochondria-dependent apoptosis of melanocytes under oxidative stress. Free Radic Biol Med.

[67] Bauer TM, Murphy E (2020). Role of mitochondrial calcium and the permeability transition pore in regulating cell death. Circ Res.

[68] Zhao S, Heng N, Wang H, Wang H, Zhang H, Gong J (2022). Mitofusins: from mitochondria to fertility. Cell Mol Life Sci.

[69] Bause AS, Haigis MC (2013). SIRT3 regulation of mitochondrial oxidative stress. Exp Gerontol.

[70] Tatone C, Di Emidio G, Barbonetti A, Carta G, Luciano AM, Falone S (2018). Sirtuins in gamete biology and reproductive physiology: emerging roles and therapeutic potential in female and male infertility. Hum Reprod Update.

[71] Zhang L, Han L, Ma R, Hou X, Yu Y, Sun S (2015). Sirt3 prevents maternal obesity-associated oxidative stress and meiotic defects in mouse oocytes. Cell cycl.

[72] Fu H, Wada-Hiraike O, Hirano M, Kawamura Y, Sakurabashi A, Shirane A (2014). SIRT3 positively regulates the expression of folliculogenesis- and luteinization-related genes and progesterone secretion by manipulating oxidative stress in human luteinized granulosa cells. Endocrinology.

[73] Kim TS, Jin YB, Kim YS, Kim S, Kim JK, Lee HM (2019). SIRT3 promotes antimycobacterial defenses by coordinating mitochondrial and autophagic functions. Autophagy.

[74] Pillai VB, Sundaresan NR, Kim G, Gupta M, Rajamohan SB, Pillai JB (2010). Exogenous NAD blocks cardiac hypertrophic response via activation of the SIRT3-LKB1-AMP-activated kinase pathway. J Biol Chem.

[75] Duan WJ, Li YF, Liu FL, Deng J, Wu YP, Yuan WL (2016). A SIRT3/AMPK/autophagy network orchestrates the protective effects of trans-resveratrol in stressed peritoneal macrophages and RAW 264.7 macrophages. Free Radic Biol Med.

[76] Li M, Li CM, Ye ZC, Huang J, Li Y, Lai W (2020). Sirt3 modulates fatty acid oxidation and attenuates cisplatin-induced AKI in mice. J Cell Mol Med.

[77] Trefts E, Shaw RJ (2021). AMPK: restoring metabolic homeostasis over space and time. Mol Cell.

[78] Li SX, Li C, Pang XR, Zhang J, Yu GC, Yeo AJ (2021). Metformin attenuates silica-induced pulmonary fibrosis by activating autophagy via the AMPK-mTOR signaling pathway. Front Pharmacol.

[79] Lawrence J, Nho R (2018). The role of the mammalian target of rapamycin (mTOR) in pulmonary fibrosis. Int J Mol Sci.

[80] Zhang M, Deng YN, Zhang JY, Liu J, Li YB, Su H (2018). SIRT3 protects rotenone-induced injury in SH-SY5Y cells by promoting autophagy through the LKB1-AMPK-mTOR pathway. Aging Dis.

[81] Marek-Iannucci S, Ozdemir AB, Moreira D, Gomez AC, Lane M, Porritt RA (2021). Autophagy-mitophagy induction attenuates cardiovascular inflammation in a murine model of Kawasaki disease vasculitis. JCI insight.

