# Supplementary material for: Sirtuin 3 regulation: a target to alleviate β-hydroxybutyric acid-induced mitochondrial dysfunction in bovine granulosa cells
Source: J Anim Sci Biotechnol. 2023 Feb 14;14:18. doi: 10.1186/s40104-022-00825-w (PMC9926763; doi:10.1186/s40104-022-00825-w)
Supplement: Supplementary file 1 — Additional file 1: Table S1. Antibody information. [file 40104_2022_825_MOESM1_ESM.docx]

**Additional file 1**

**Table S1** Antibody information

| **Antibody name** | **Dilution ratio** | **Source** | **Cat. #** |
| --- | --- | --- | --- |
| FSHR | 1:200 | Proteintech | 22665-1-AP |
| BAX | 1:5000 | Proteintech | 50599-2-Ig |
| BCL2 | 1:1000 | Proteintech | 12789-1-AP |
| Caspase3 | 1:1000 | Proteintech | 19677-1-AP |
| Caspase9 | 1:1000 | Proteintech | 10380-1-AP |
| Sirt3 | 1:1000 | Proteintech | 10099-1-AP |
| p-AMPK | 1:1000 | Cell Signaling Technology (CST) | 2535S |
| AMPK | 1:1000 | Cell Signaling Technology (CST) | 5831S |
| p-mTOR | 1:1000 | Affinity | AF3308 |
| mTOR | 1:1000 | Affinity | AF6308 |
| Beclin-1 | 1:1000 | Proteintech | 11306-1-AP |
| β-Actin | 1:1000 | Cell Signaling Technology (CST) | 4970S |
| Goat Anti Rabbit IgG (H+L)  -DyLight 488 | 1:1000 | Gene-Protein Link | P03S06S |
| Anti-rabbit IgG HRP-linked Antibody | 1:2000 | Cell Signaling Technology (CST) | 7074S |
