# Supplementary material for: Sirtuin 3 regulation: a target to alleviate β-hydroxybutyric acid-induced mitochondrial dysfunction in bovine granulosa cells
Source: J Anim Sci Biotechnol. 2023 Feb 14;14:18. doi: 10.1186/s40104-022-00825-w (PMC9926763; doi:10.1186/s40104-022-00825-w)
Supplement: Supplementary file 2 — Additional file 2: Table S2. Nucleotide information. [file 40104_2022_825_MOESM2_ESM.docx]

**Additional file 2**

**Table S2** Nucleotide information

| **Primer name** | **GenBank accession No.** | **Sequence 5'→3'** | **PCR size, bp** | **Tm, ℃** | **Application** |
| --- | --- | --- | --- | --- | --- |
| *Mn-SOD*-Forward | NM_201527.2 | CCTGCAAGGAACAACAGGTCT | 104 | 60 | RT-PCR |
| *Mn-SOD*-Reverse |  | TGGCCTTCAGATAATCGGGC |  |  |  |
| *OPA1*-Forward | NM_001192961.1 | GCCTGACATTGTGTGGGAGA | 160 | 60 | RT-PCR |
| *OPA1*-Reverse |  | TCCAGGTGAACCTGTGGTG |  |  |  |
| *FIS1*-Forward | NM_001034784.2 | GCGTCTGAGTTGTGAGACCT | 180 | 60 | RT-PCR |
| *FIS1*-Reverse |  | CGTTGTACTTGCTTCGCACC |  |  |  |
| *Sirt3*-Forward | XM_005200933.4 | TCCTCCGATTGCTACACGAG | 187 | 60 | RT-PCR |
| *Sirt3*-Reverse |  | GCAAAAGGTTCCACCCAGAAGT |  |  |  |
| *β-Actin*-Forward | NM_173979.3 | GCCCTGAGGCTCTCTTCCA | 101 | 60 | RT-PCR |
| *β-Actin*-Reverse |  | GCGGATGTCGACGTCACA |  |  |  |
| si-Sirt3-Forward | XM_005200933.4 | GGUGGAGGAUGGUCCAUAUTT |  |  | siRNA |
| si-Sirt3-Reverse |  | AUAUGGACCAUCCUCCACCTT |  |  |  |
