# Supplementary figures and images for: Sirtuin 3 regulation: a target to alleviate β-hydroxybutyric acid-induced mitochondrial dysfunction in bovine granulosa cells
Source: J Anim Sci Biotechnol. 2023 Feb 14;14:18. doi: 10.1186/s40104-022-00825-w (PMC9926763; doi:10.1186/s40104-022-00825-w)

**Additional file 3**


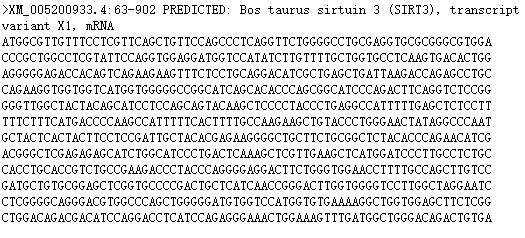


**Fig. S1** The specific sequences of the *Sirt3* overexpression plasmid

Supplement: Supplementary file 3 — Additional file 3: Fig. S1. The specific sequences of the Sirt3 overexpression plasmid. [file 40104_2022_825_MOESM3_ESM.docx]
