# Supplementary material for: Sirtuin 3 regulation: a target to alleviate β-hydroxybutyric acid-induced mitochondrial dysfunction in bovine granulosa cells
Source: J Anim Sci Biotechnol. 2023 Feb 14;14:18. doi: 10.1186/s40104-022-00825-w (PMC9926763; doi:10.1186/s40104-022-00825-w)
Supplement: Supplementary file 4 — Additional file 4: Fig. S2. Relative expression analysis of mitochondrial fusion (OPA1) and division(FIS1)-related genes. *P < 0.05; **P < 0.01. [file 40104_2022_825_MOESM4_ESM.docx]

**Additional file 4**


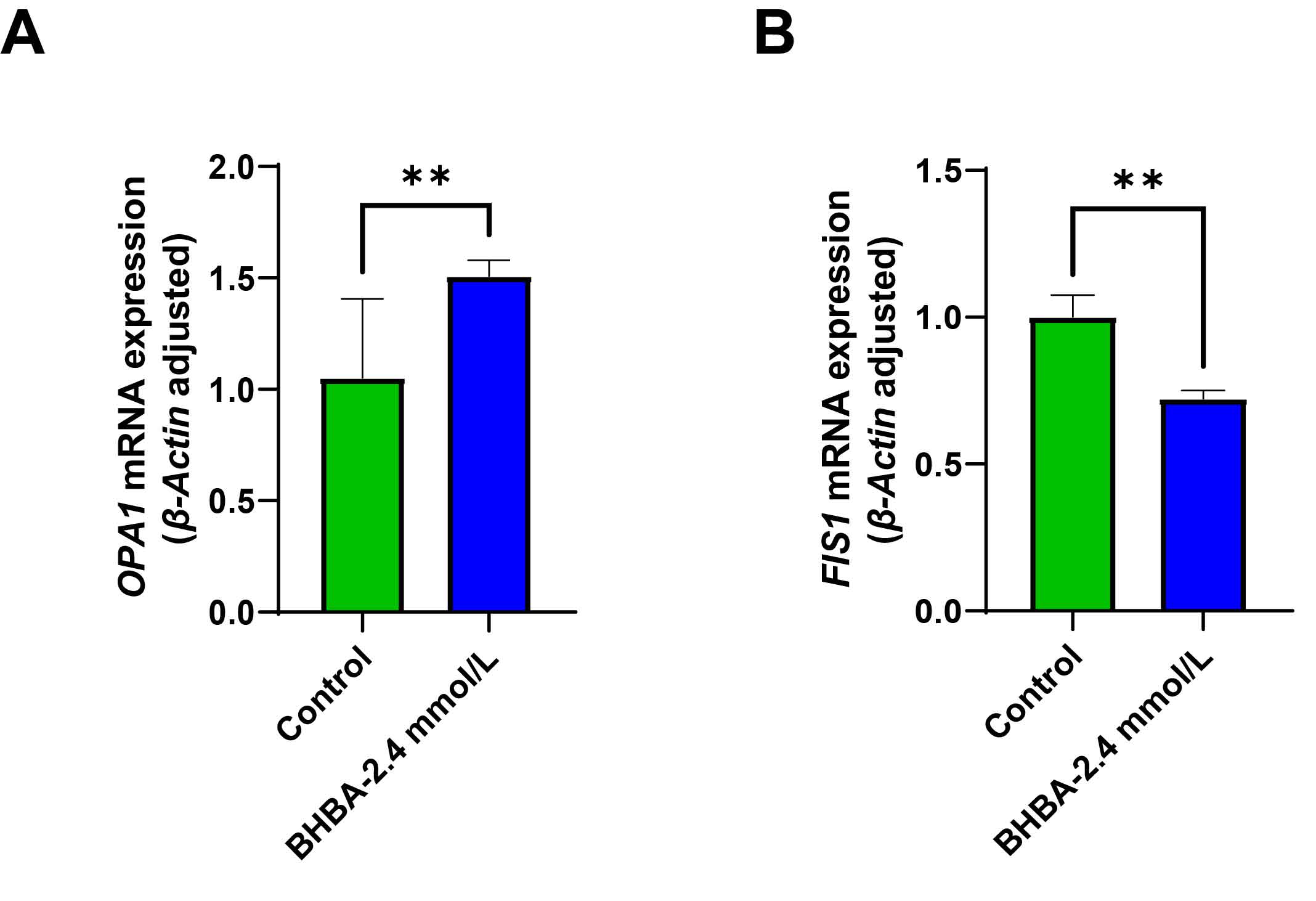


**Fig. S2** Relative expression analysis of mitochondrial fusion (*OPA1*) and division (*FIS1*)-related genes. The significant difference analysis is performed by using Student *t*-tests, ^**^*P* < 0.01
