# Supplementary material for: Sirtuin 3 regulation: a target to alleviate β-hydroxybutyric acid-induced mitochondrial dysfunction in bovine granulosa cells
Source: J Anim Sci Biotechnol. 2023 Feb 14;14:18. doi: 10.1186/s40104-022-00825-w (PMC9926763; doi:10.1186/s40104-022-00825-w)
Supplement: Supplementary file 5 — Additional file 5: Fig. S3. The effect of Sirt3 knockdown on the proliferation and apoptosis of fGCs. (A) Assay showing siRNA interference efficiency (B) Analysis of theproliferation rate of fGCs after Sirt3 knockdown for 24 h, 48 and72 h. (C-D) Analysis of the apoptosis rate of fGCs after Sirt3 knockdown for72 h. *P < 0.05; **P < 0.01. [file 40104_2022_825_MOESM5_ESM.docx]

**Additional file 5**


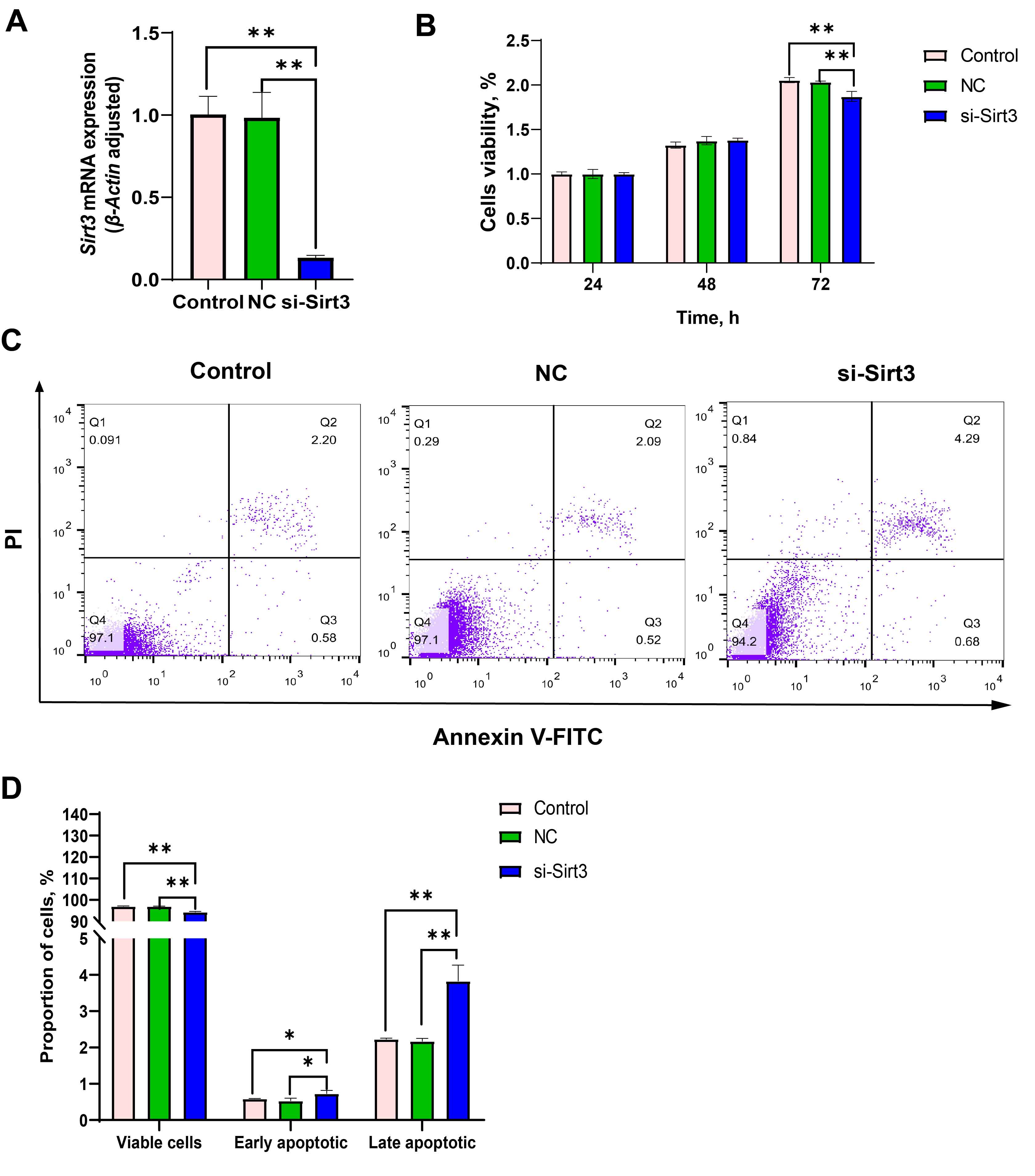


**Fig. S3** The effect of *Sirt3* knockdown on the proliferation and apoptosis of fGCs. (**A**) Assay showing siRNA interference efficiency (**B**) Analysis of the proliferation rate of fGCs after *Sirt3* knockdown for 24 h, 48 h and 72 h. (**C-D**) Analysis of the apoptosis rate of fGCs after *Sirt3* knockdown for 72 h. The significant difference analysis is performed by using one-way ANOVA, ^*^*P* < 0.05, ^**^*P* < 0.01
